# Rapid four-component synthesis of dihydropyrano[2,3-*c*]pyrazoles using nano-eggshell/Ti(IV) as a highly compatible natural based catalyst

**DOI:** 10.1186/s13065-021-00734-5

**Published:** 2021-01-25

**Authors:** Arefeh Dehghani Tafti, Bi Bi Fatemeh Mirjalili, Abdolhamid Bamoniri, Naeimeh Salehi

**Affiliations:** 1 Department of Chemistry, College of Science, Yazd University, P.O.Box 89195-741, Yazd, I.R. of Iran; 2 Department of Organic Chemistry, Faculty of Chemistry, University of Kashan, Kashan, I.R. of Iran

**Keywords:** Dihydropyrano[2,3-c]pyrazole, Nano-eggshell/Ti(IV), Multicomponent reactions, Eggshell, Titanium tetrachloride, Natural catalyst

## Abstract

Nano-eggshell/Ti(IV) as a novel naturally based catalyst was prepared, characterized and applied for the synthesis of dihydropyrano[2,3-*c*]pyrazole derivatives. The characterization of nano-eggshell/Ti(IV) was performed using Fourier Transform Infrared spectroscopy, X-ray Diffraction, Field Emission Scanning Electron Microscopy, Energy-Dispersive X-ray Spectroscopy, and Thermo Gravimetric Analysis. Dihydropyrano[2,3-*c*]pyrazoles were synthesized in the presence of nano-eggshell/Ti(IV) via a four component reaction of aldehydes, ethyl acetoacetate, malononitrile and hydrazine hydrate at room temperature under solvent free conditions. The principal affairs of this procedure are mild condition, short reaction times, easy work-up, high yields, reusability of the catalyst and the absence of toxic organic solvents.
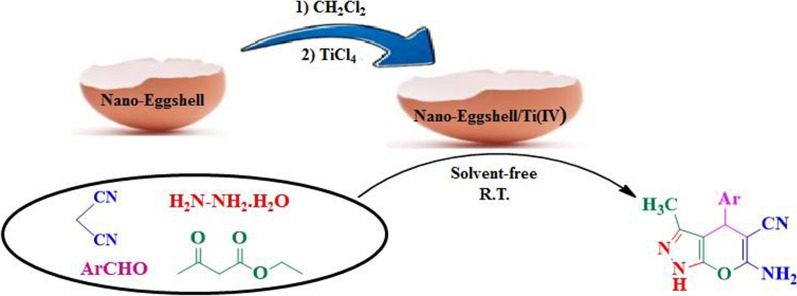

## Introduction

One key-step toward green chemistry concerns on chemical transformations under solvent-free conditions [1, 2]. Solvent free conditions often have lead to decrease reaction time, increase yields and easy work-up [3, 4]. Combining this condition with multicomponent reactions (MCRs) disclosed a particular opportunity for architecting of heterocyclic molecules in short time [5, 6]. MCRs play an essential role in combinatorial chemistry due to one-pot synthesis of various complex molecules, atom economy and effectiveness compared with single step reaction [7, 8]. For economic and environmental reasons, solvent free reactions were demonstrated to be an efficient method for the synthesis of chemical product in a clean and safe conditions [9–11]. Dihdropyrano[2,3-*c*]pyrazoles (DHPPs) are important class of heterocycle componds because of their wide applications in medicinal and pharmaceutical chemistry [12]. Many of these properties are known for their anti-microbial [13], anti-inflammatory [14], anti-cancer [15], bactericidal [16], molluscicida [17], and kinase inhibitory [18] activities. In the first report, DHPP was synthesized by a reaction between 3-methyl-1-phenylpyrazolin-5-one and tetracyanoethylene [19]. Recently, DHPPs have been synthesized via the reaction of hydrazine hydrate, ethyl acetoacetate, malononitrile, and aldehydes. Some catalysts have been used to develop the above mentioned reaction such as γ-alumina [20], glycine [21], ionic liquids [22], l-proline [23], imidazole [24], I_2_ [25], and trietheylamine [26]. In the recent years, heterogeneous catalysts, due to the high capability for recycling and reutility, have surpassed homogeneous catalytic systems, despite their benefits such as high activity and selectivity [27]. Nowadays, nanocatalysts have been subjected of immense interest, because of their potential applications in different fields. They have several important advantages as heterogeneous catalysts including high catalytic activity, readily available, simple separation, high degree of chemical stability, and reusability [28–31].

The eggshell is represented 11% of the total weight of the egg and composed predominantly of calcium carbonate (94%), organic materials (4%), calcium phosphate (1%), and magnesium carbonate (1%) [32].

**Scheme 1.**
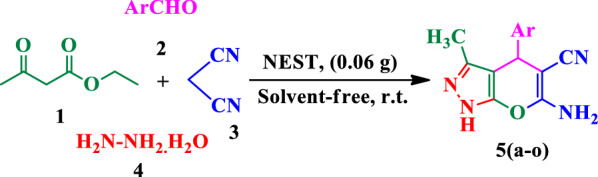
Synthesis of dihydropyrano[2,3-*c*]pyrazoles catalyzed by nano-eggshell/Ti(IV)

In continuation of our previous works in using solid acid catalysts [33–38], herein, we reporte an efficient one-pot four-component reaction protocol for the synthesis of DHPPs in the presence of nano-eggshell/Ti(IV) (NEST) as a highly effective nanocatalyst in good to excellent yields under mild conditions (Scheme 1).

## Results and discussion

### Characterization of the nanocatalyst

**Scheme 2.**
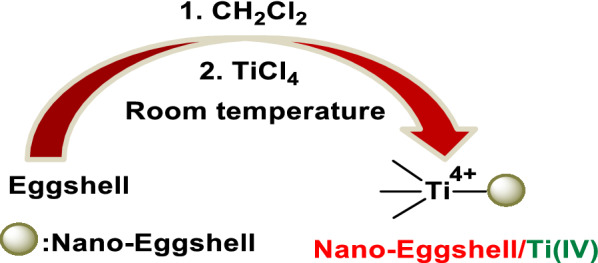
Preparation of NEST

NEST was prepared simply via addition of TiCl_4_ to a suspension of eggshell nanoparticles in CH_2_Cl_2_ (Scheme 2). The obtained catalyst was characterized using Fourier Transform Infrared (FT-IR) spectroscopy, X-ray Diffraction (XRD), Field Emission Scanning Electron Microscopy (FESEM), Energy-Dispersive X-ray (EDX) spectroscopy, and Thermo Gravimetric Analysis (TGA).

**Fig. 1:**
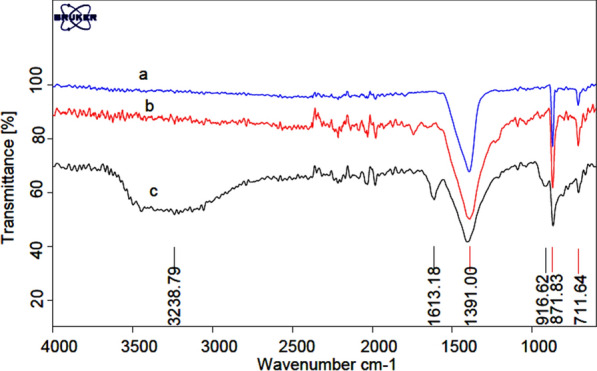
FT-IR spectra of **a** CaCO_3_, **b** nano-eggshell, and **c** NEST

The FT-IR spectra of CaCO_3_ [39, 40], nano-eggshell, and NEST are shown in Fig. 1. Distinct absorption bands can be observed at 711, 871, and 1391 cm^−1^ in all compared spectra show the presence of high percentage of CaCO_3_ in eggshell and NEST. For NEST (Fig. 1c), in addition to the eggshell absorption bands, stretching vibrations of C–O–Ti group at 780 cm^−1^ (according to previously reported FT-IR about Ti(OBut)_4_ [41, 42]) was appeared, indicated that TiCl_4_ have functionalized on nano-eggshell successfully. The absorbed band at 1613 cm^−1^ is associated to the bending vibration of H–O–H which have shown the absorbed water on catalyst [43].

**Fig. 2:**
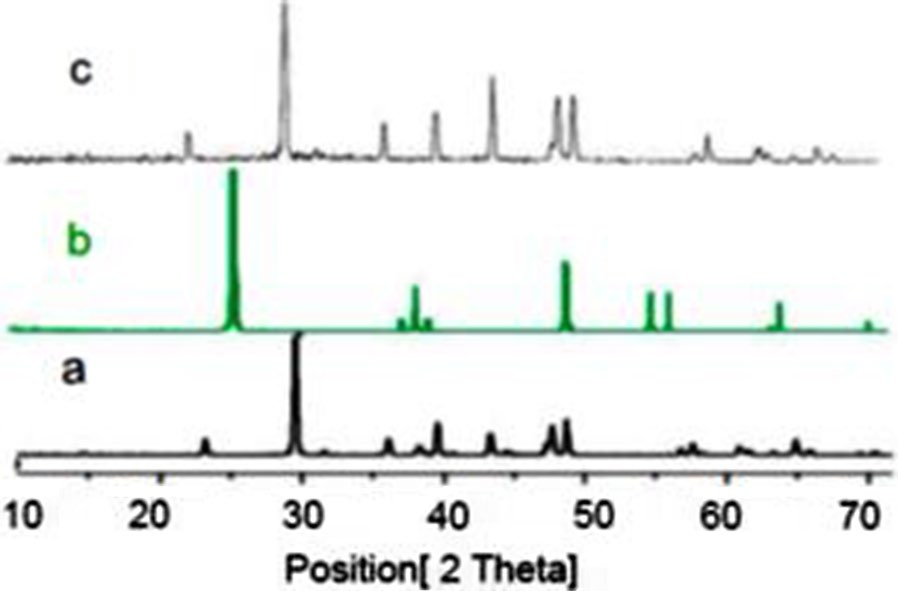
The XRD patterns of **a** CaCO_3_, **b** TiO_2_, and **c** NEST

Figure 2 shows the XRD patterns of NEST, TiO_2_ and CaCO_3_ in the range of 10–70° (2θ). NEST (Fig. 2c), has shown diffraction peaks at 2θ = 23, 29, 37, 40, 43, 47, 48, 56, 57, 61 and 62°, which are quite matched with the structure of pure CaCO_3_. By comparison with Fig. 2a–c, we can conclude the absence of TiO_2_ and the presence of CaCO_3_ in catalyst.

**Fig. 3:**
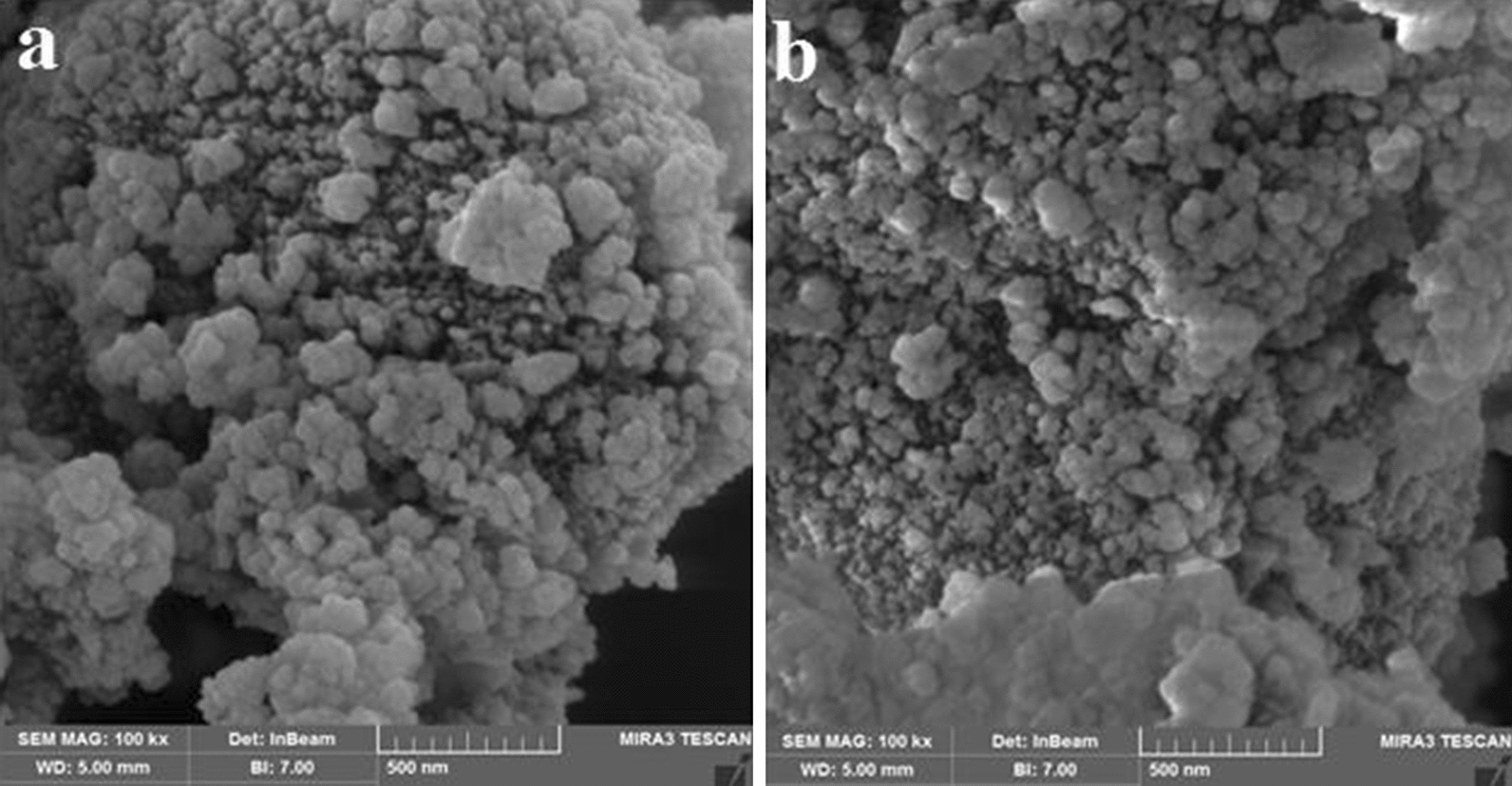
FESEM image of **a** nano-eggshell, **b** NEST

Surface morphology of nano-eggshell and the synthesized NEST was observed using FESEM analysis (Fig. 3a, b). The FESEM image of NEST (Fig. 3b) indicates that morphology of the nano particles has a quasi-spherical shape. The average size of NEST was estimated about 40 nm.

**Fig. 4:**
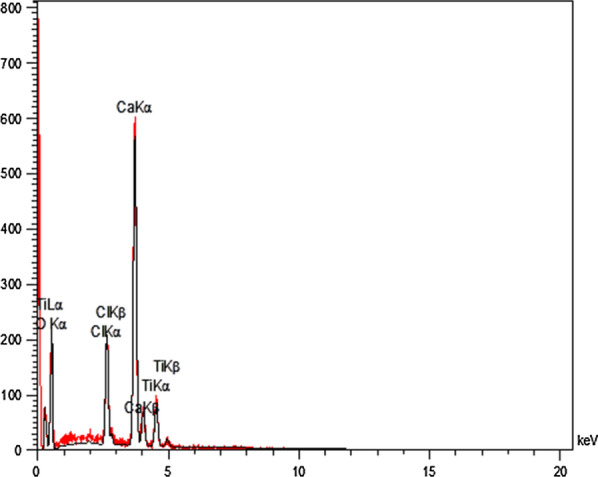
EDS analysis of NEST

The existence of expected elements in the structure of the NEST was approved by EDX analysis (Fig. 4). The EDX results have clearly confirmed the presence of C, O, Cl, Ca and Ti in the catalyst. According to this data, the weight percentages of the above-mentioned elements are 14.48, 43.13, 7.16, 29.30 and 5.94, respectively.

**Fig. 5:**
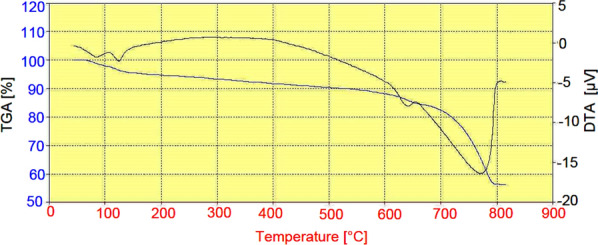
TGA and DTA patterns of NEST

For thermal stability investigation of the catalyst, TGA-DTA analysis was done in a range of 45–813 °C (Fig. 5). The first decrease of weight was assigned to the catalyst moisture removal (endothermic effect at 70–130 °C, 4% weight loss). The second weight loss (16%) was occurred at 130–600 °C with an exothermic process. As the temperature increased to 800 °C, the main mass loss could be associated with the decomposition of eggshell to CO_2_ and CaO.

To optimize the conditions for the synthesis of the DHPPs in the presence of NEST, the condensation of 4-chlorobenzaldehyde, malononitrile, ethyl acetoacetate, and hydrazine hydrate in the molar ratio 1:1:1:2 was done under various conditions (Table 1). According to the obtained data, the best yield of 6-amino-4-(4-chlorophenyl)-3-methyl-1,4-dihydropyrano[2,3-*c*]pyrazole-5-carbonitrile (**5h**) was achieved using 0.06 g of NEST at room temperature under solvent-free condition (Table 1, entry 12).

**Table 1:** Preparation of 5 h in the presence of NEST under various conditions

Entry	Conditions	Time (min)	Yield^a^ (%)
	Solvent/catalyst (g)/Temp. (°C)		
1	H_2_O/NEST (0.06)/r.t	180	55
2	H_2_O/NEST (0.06)/Reflux	120	58
3	EtOH/NEST (0.06)/r.t	60	75
4	EtOH/NEST (0.06)/Reflux	60	80
5	H_2_O:EtOH (1:1)/NEST (0.06)/r.t	45	83
6	H_2_O:EtOH (1:1)/NEST (0.06)/Reflux	30	85
7	–/NEST (0.06)/35	90	77
8	–/NEST (0.06)/60	150	70
9	–/–/r.t	30	25
10	–/NEST (0.02)/r.t	45	85
11	–/NEST (0.04)/r.t	20	90
12	–/NEST (0.06)/r.t	15	94
13	–/NEST (0.1)/r.t	20	89

Reaction was performed with ethyl acetoacetate (1 mmol), 4-chlorobenzaldehyde (1 mmol), malononitrile (1 mmol), and hydrazine hydrate (2 mmol)

^a^Isolated yield

After optimization of the reaction conditions for preparation of DHPPs, various aromatic and heteroaromatic aldehydes were used for expansion of this protocol. The reactions were proceeded for all used aldehydes (Table 2). The desired products were isolated in good to excellent yields in short reaction times without any byproducts.

**Table 2:** Synthesis of DHPPs **5**(**a**–**o**) in the presence of NEST

Entry	Ar	Product	Time (min)	Yield^a^ (%)	Mp (°C)	Refs.
1	C_6_H_5_	**5a**	10	92	242–244	[44]
2	2-OCH_3_C_6_H_4_	**5b**	15	89	226–228	[45]
3	3-O_2_NC_6_H_4_	**5c**	10	90	210–211	[45]
4	4-H_3_CC_6_H_4_4	**5d**	18	87	204–206	[46]
5	4-O_2_NC_6_H_4_	**5e**	9	93	239–242	[45]
6	3-BrC_6_H_4_	**5f**	15	94	223–224	[46]
7	4-BrC_6_H_4_	**5g**	12	96	178–180	[45]
8	4-ClC_6_H_5_	**5 h**	10	94	230–232	[44]
9	4-OHC_6_H_4_	**5i**	10	95	222–224	[46]
10	3,4-(OH)C_6_H_3_	**5j**	10	91	225–227	[47]
11	2,4-(Cl)C_6_H_3_	**5k**	20	90	223–225	[46]
12	3-OCH_3_ 4-OH, C_6_H_3_	**5l**	15	92	234–236	[46]
13	4-FC_6_H_4_	**5m**	8	96	212–214	[45]
14	2-Furyl	**5n**	10	91	228–230	[48]
15	1-Naphthyl	**5o**	25	88	206–208	[20]

^a^Isolated yield

**Scheme 3.**
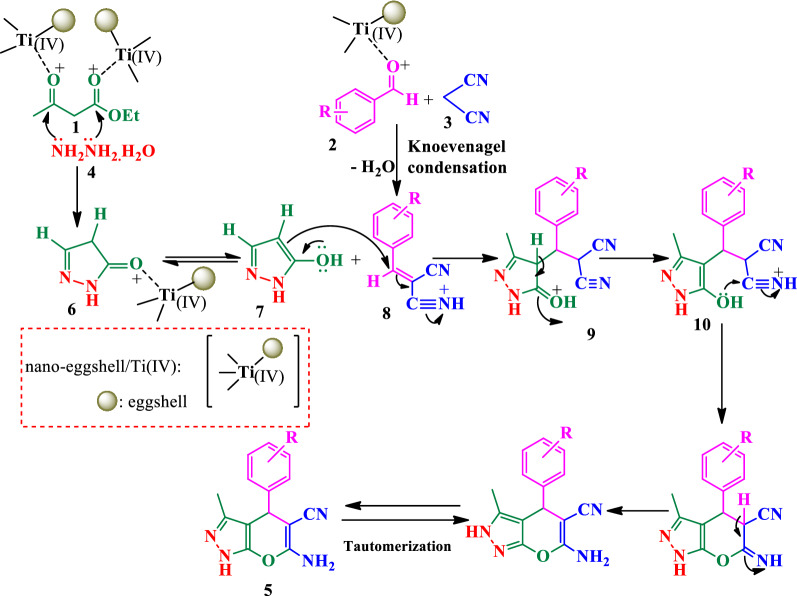
Proposed mechanism for the synthesis of DHPPs

A proposed mechanism for the synthesis of DHPPs catalyzed by NEST was shown in Scheme 3. Initially, the condensation of hydrazine hydrate (**4**) and ethyl acetoacetate (**1**) was formed intermediate (**6**) in the presence of NEST as a Lewis acid. The Knoevenagel condensation of malononitrile (**3**) with aromatic aldehyde (**1**) was produced the intermediate (**8**). Michael addition reaction of the intermediate (**8**) and (**7**) were generated intermediate (**10**), followed by intramolecular cyclization and tautomerization have given the DHPPs (**5**).

**Fig. 6:**
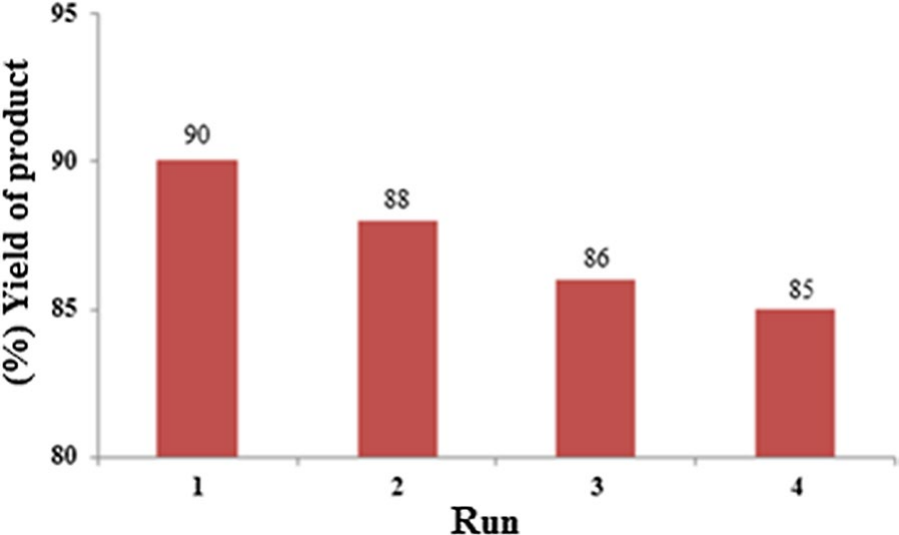
Reusability of NEST

In order to investigation of the catalyst reusability, after the reaction completion, the NEST was isolated by adding acetone to reaction mixture and then filtered. The recovered catalyst was washed with dichloromethane and dried at room temperature. It was observed that the recovered nanocatalyst could be used at least four times without significant loss of its catalytic activity (Fig. 6).

**Fig. 7:**
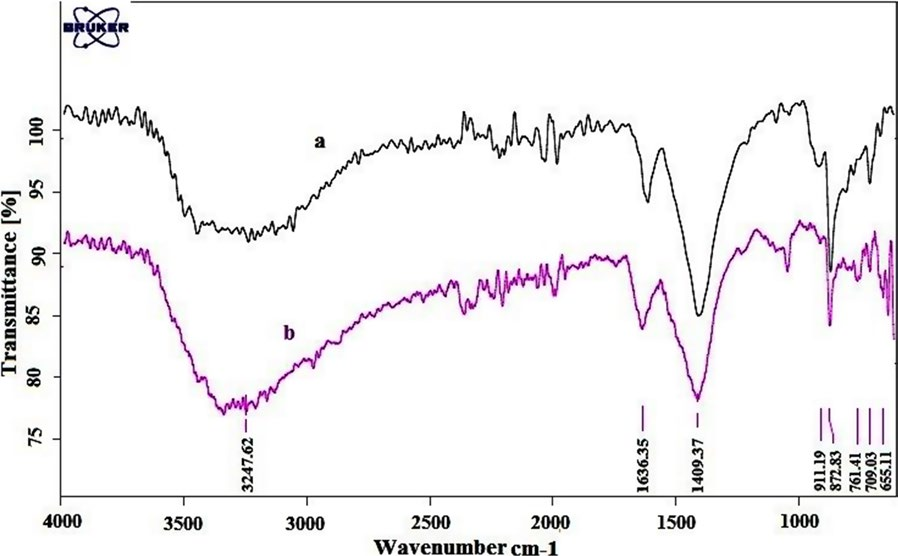
FT-IR spectrum of **a** fresh NEST, **b** recovered NEST

**Fig. 8:**
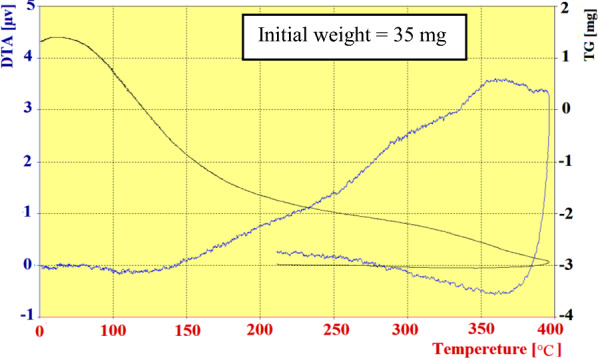
TGA and DTA results of recovered NEST

The structure of recovered catalyst was studied by FT-IR (Fig. 7) and TGA-DTA (Fig. 8). The comparison between fresh and recoverable catalysts have shown no differences.

Finally, the catalytic performance of NEST was compared with that of other previously reported catalysts for the synthesis of 5a (Table 3). From the viewpoints of green chemistry and simplicity, our method is a good one.

**Table 3:** Catalytic performances of NEST in comparison with some other catalysts for synthesis of **5a**

Entry	Catalyst	Solvent	Temp (°C)	Time (min)	Yield^a^ (%) [Refs.]
1	I_2_	H_2_O	r.t	10	90 [49]
2	Isonicotinic acid	–	80	10	92 [50]
3	Piperidine	H_2_O	r.t	5–10	89 [51]
4	γ-Alumina	H_2_O	Reflux	35	90 [20]
5	Et_3_N	EtOH	Reflux	15	72 [26]
6	NEST^b^	–	r.t	10	92

^a^Isolated yield

^b^This work

## Conclusion

In this work, we have synthesized the NEST and characterized it as a novel heterogeneous natural nanocatalyst. This catalyst was used for the synthesis of DHPPs at room temperature under solvent free condition via condensation of hydrazine hydrate, ethyl acetoacetate, malononitrile, and aromatic aldehydes. This method includes some advantages such as the solvent-free condition, good to excellent yields, room temperature, short reaction time, easy work-up and reusability of the catalyst.

## Experimental section

### Chemicals and apparatus

All compounds were purchased from Merck, Aldrich and Fluka chemical companies. FT-IR spectra were run on a Bruker, Equinox 55 spectrometer. A Bruker (DRX-400 Avance) NMR was used to record the ^1^H and ^13^C NMR spectra. The morphology of the particles was observed by FESEM under acceleration voltage of 120 kV. The XRD patterns were obtained on a Philips Xpert MPD diffractometer (Cu Ka, radiation, k¼ 0.154056 nm). EDS was obtained using a Phenom pro X instrument. TGA was conducted using STA 504 instrument.

### Preparation of NEST

Firstly, the eggshell was heated in boiling water for 30 min, dried in oven 150 °C and powdered. Then, 1 g of prepared nano-eggshell powder was stirred for 30 min in 10 mL of dried CH_2_Cl_2_. Titanium tetrachloride (4.36 mL) was slowly added dropwise to the mixture. After stirring at room temperature for 30 min, the resulting product filtered and washed with dichloromethane three times. Finally, the obtained NEST was dried at room temperature for 3 h.

### General procedure for the synthesis of DHPPs

In a 100 mL round bottom flask, a mixture of aldehyde (1 mmol), malononitrile (1 mmol), hydrazine hydrate (2 mmol), ethyl acetoacetate (1 mmol) and NEST (0.06 g) was stirred at room temperature. Progress of the reaction was monitored by TLC (*n*-hexane:EtOAc, 4:1). After completion of the reaction, the mixture was dissolved in acetone. Then, the catalyst was filtered off and the obtained solution was poured into cold water. The obtained solid product was filtered and purified by recrystallization from ethanol and water (4:1). The obtained NEST catalyst was then washed with EtOH, dried and reused directly for four times in other fresh reactions with negligible decreasing of the yields.

### Spectroscopic data for some products

#### 6-Amino-3-methyl-4-(3-nitrophenyl)-1,4-dihydropyrano[2,3-*c*]pyrazole-5-carbonitrile (Table 2, entry 3)

White solid. M.P. 210–211 °C FT-IR (ATR)/ῡ (cm^−1^): 3484, 3231, 3120, 2190, 1645, 1597, 1519, 1491, 1410, 1351, 733. ^1^H NMR (400 MHz, DMSO-d_6_)/δ (ppm): 1.82 (s, 3H), 4.89 (s, 1H), 7.08 (s, 2H), 7.64–7.70 (m, 2H), 8.04 (s, 1H), 8.13–8.15 (d, *J* = 8 Hz, 1H), 12.23 (s, 1H).; ^13^C NMR (100 MHz, DMSO-d_6_)/δ ppm: 161.63, 155.17, 148.36, 147.32, 136.38, 134.88, 130.47, 122.33, 121.01, 97.15, 56.59, 36.11, 10.25.

#### 6-Amino-3-methyl-4-(4-nitrophenyl)-1,4-dihydropyrano[2,3-c]pyrazole-5-carbonitrile (Table 2, entry 5)

White solid. M.P. 239–242 °C. FT-IR (ATR)/ῡ (cm^−1^): 3475, 3227, 3106, 2195, 1646, 1592, 1513, 1399, 1348, 1163, 1109, 810, 744; ^1^H NMR(400 MHz, Acetone-d_6_)/δ ppm: 2 (s, 3H), 4.88 (s, 1H), 6.30 (br s, 2H), 7.55 (d, *J* = 8 Hz, 2H), 8.23 (d, *J* = 8 Hz, 2H), 11.43 (s, 1H). ^13^C NMR (100 MHz, DMSO-d_6_)/δ ppm: 161.62, 155.15, 152.59, 146.85, 136.36, 132.19, 129.32, 124.38, 120.98, 97.04, 56.37, 36.36, 10.22.

#### 6-Amino-4-(4-hydroxyphenyl)-3-methyl-1,4-dihydropyrano[2,3-c]pyrazole-5-carbonitrile (Table 2, entry 9)

White solid. M.P. 222–224 °C. FT-IR (ATR)/ῡ (cm^−1^): 3372, 3304, 3127, 2173, 1645, 1594, 1510, 1489, 1441, 1404, 1189, 1166, 1041, 809. ^1^H NMR (400 MHz, Acetone-d_6_)/δ(ppm): 1.74 (s, 3H), 4.44 (s, 1H), 6.65 (dd, *J* = 7.5 Hz, *J* = 3.7 Hz, 2H), 6.76 (br s, 2H), 6.91 (dd, *J* = 7.5 Hz, *J* = 3.7 Hz, 2H), 9.27 (s, 1H), 12.02 (s, 1H).; ^13^C NMR (100 MHz, DMSO-d_6_)/δ ppm: 161.10, 156.49, 155.22, 135.98, 135.24, 128.92, 121.40, 115.58, 98.54, 58.21, 35.95, 10.24.

#### 6-Amino-4-(2,4-dichlorophenyl)-3-methyl-1,4-dihydropyrano[2,3-c]pyrazole-5-carbonitrile (Table 2, entry 11)

Pale yellow solid. M.P. 223–225 °C. FT-IR (ATR)/ῡ (cm^−1^): 3482, 3243, 3115, 2186, 1638, 1587, 1491, 1408, 1100, 1052, 866, 741.; ^1^H NMR (400 MHz, DMSO-d_6_)/δ ppm: 1.85 (s, 3H), 5.13 (s, 1H), 7.07 (s, 2H), 7.29 (d, *J* = 8 Hz, 1H), 7.47 (dd, *J* = 8.4 Hz, *J* = 2 Hz, 1H), 7.65 (d, *J* = 2.4 Hz, 1H), 12.23 (s, 1H).; ^13^C NMR (100 MHz, DMSO-d_6_)/δ ppm: 161.30, 154.88, 140.07, 135.44, 132.81, 132.10, 128.83, 128.02, 120.25, 96.32, 55.21, 33.07, 9.53.

## Data Availability

All data generated or analysed during this study are included in this published article.

## Abbreviations

NEST: Nano-eggshell/Ti(IV)
MCRs: Multi-component reactions
EtOH: Ethanol
FESEM: Field Emission Scanning Electron Microscope
FT-IR: Fourier Transform Infrared
XRD: X-ray diffraction
EDX: Energy-Dispersive X-ray
TGA: Thermo Gravimetric Analysis
NMR: Nuclear magnetic resonance
TLC: Thin layer chromatography
